# HPV Vaccination in Immunosuppressed Patients with Established Skin Warts and Non-Melanoma Skin Cancer: A Single-Institutional Cohort Study

**DOI:** 10.3390/vaccines11091490

**Published:** 2023-09-15

**Authors:** Simon Bossart, Cloé Daneluzzi, Matthias B. Moor, Cédric Hirzel, Kristine Heidemeyer, S. Morteza Seyed Jafari, Robert E. Hunger, Daniel Sidler

**Affiliations:** 1 Department of Dermatology, Inselspital University Hospital of Bern, University of Bern, 3010 Bern, Switzerland; cloe.daneluzzi@h-ju.ch (C.D.); seyedjafarism@yahoo.com (S.M.S.J.); robert.hunger@insel.ch (R.E.H.); 2 Department of Nephrology, Inselspital University Hospital of Bern, University of Bern, 3010 Bern, Switzerland; matthias.moor@unibe.ch (M.B.M.); daniel.sidler@insel.ch (D.S.); 3 Department of Infectious Diseases, Inselspital University Hospital of Bern, University of Bern, 3010 Bern, Switzerland; cedric.hirzel@insel.ch

**Keywords:** HPV, human papillomavirus, nonavalent, human papillomavirus vaccine, non-melanoma skin cancer, organ transplantation, immunosuppression

## Abstract

cSCC (cutaneous squamous cell carcinoma) and its precursors are a major cause of morbidity, especially in immunosuppressed patients, and are frequently associated with human papillomavirus (HPV) infections. The purpose of this study is to investigate the therapeutic potential of alpha-HPV vaccination for immunosuppressed patients with established cSCC and its precursors. In this retrospective study, all patients who received Gardasil-9^®^, a nonavalent HPV vaccine, as secondary prophylaxis were examined. Dermatologic interventions in both the pre- and post-vaccination periods were analyzed with zero-inflated Poisson regression and a proportional intensity model for repeated events with consideration of the clinically relevant cofactors. The hazard ratio for major dermatologic interventions was 0.27 (CI 0.14–0.51, *p* < 0.001) between pre- and post-Gardasil-9^®^ intervention. Gardasil-9^®^ vaccination showed good efficacy in reducing major dermatologic interventions even after correction of relevant cofactors and national COVID-19 caseloads during the observational period. Alpha-HPV vaccination may potentially cause a significant decrease in dermatologic interventions and overall mortality as well as healthcare costs in immunosuppressed patients with high skin tumor burden.

## 1. Introduction

Skin warts and especially non-melanoma skin cancer (NMSC) are a major cause of morbidity in patients under chronic immunosuppressive treatment, such as patients after solid organ transplantation (SOT) [1,2]. The incidence of NMSC increases with the duration of immunosuppressive therapy and develops mainly in sun-exposed areas. While cutaneous squamous cell carcinoma (cSCC) is the most common skin cancer in transplant recipients, occurring 65–250 times more frequently than in the normal population, basal cell carcinoma is increased by a factor of 10 after organ transplantation [2,3]. In addition, the occurrence of NMSC is associated with the presence of multiple keratotic lesions, such as warts and premalignant actinic keratoses, as well as intraepithelial neoplasia (Bowen’s disease). These conditions can develop not only on sun-exposed keratinized skin but also on mucous membranes [3,4]. Human papillomavirus (HPV) appears to play an important co-carcinogenic role. Persistent HPV-induced skin warts, cSCC, and their precursor lesions (actinic keratosis, Bowen’s disease), which arise from Human papillomavirus (HPV) appear to play a crucial co-carcinogenic role. Persistent HPV-induced skin warts, cSCC, and their precursor lesions (actinic keratosis and Bowen’s disease), primarily arising in sun-exposed areas, constitute the major skin tumor types in immunosuppressed patients. After more than 10 years post-transplantation, up to 90% of solid organ transplant recipients (SOT) develop skin warts and NMSC, respectively [4,5]. An important observation is the association and co-localization of cSCC with HPV-induced warts in SOT, suggesting that persistent warts may progress to skin cancer. Furthermore, in these patients, warts persist over years, displaying dysplastic features. Overall, SOT individuals have a significantly higher HPV prevalence rate of up to 90% in cutaneous SCC compared to the general population (11–32%). Additionally, the number of multiple HPV types is higher in cSCC from SOT individuals compared to the general population. Consequently, due to years of immunosuppression and chronic HPV infection in solid organ transplant recipients (SOT), a significant burden of warts and NMSC accumulates. This necessitates frequent dermatological visits and is associated with substantial healthcare costs [4,5,6,7]. In HPV infections, there are different types that can affect the keratinocytes of the skin and mucosa. HPVs that affect the mucosa are classified as alpha HPVs. These are divided into low-risk types such as HPV 6 and 11, which induce most condylomas, and high-risk types such as HPV 16 and 18, which are involved in carcinogenesis of the anogenital region and oropharynx. HPVs that affect keratinized skin include the alpha-, beta-, gamma-, Mu-, and Nu-HPV genera [8].

Beta-HPV infection in keratinized skin appears to play a predisposing role in the genesis of NMSC and has been significantly clustered in cSCCs of immunosuppressed transplant recipients. The carcinogenic effect is seen as a combination of beta-HPV infection with ultraviolet (UV) light [9,10].

To prevent HPV infection, only vaccines for alpha-HPV infection have been developed and licensed. Vaccines for the prevention of beta-HPV strains are currently still under investigation [10]. The three licensed HPV vaccines (Cervarix^®^, Gardasil^®^, and Gardasil-9^®^) on the market are designed to prevent α-HPV-related diseases and provide highly effective protection specific to oral and anogenital mucosal HPV subtypes [10,11]. The target of this vaccine is the viral capsid L1 of specific alpha HPV strains in mucosal types. Alpha-HPV and beta-HPV, however, show similarities in the immunogenic L1 and L2 capsid proteins [10,12]. In this regard, there is anecdotic evidence that HPV vaccination against alpha-genera has also resulted in the regression of cutaneous warts and cSCCs [13,14,15,16]. The similar expression of capsid proteins within the alpha and beta HPV genera could be a possible explanation in this regard. Furthermore, mixed infections of alpha and beta HPV are discussed, explaining the response of vaccination against alpha strains [10,12,13,14,15,16].

However, no definitive explanations or systematic studies exist to investigate the therapeutic potential of alpha-HPV vaccination for NMSC or its precursors and warts.

This work evaluates the recent activity of HPV immunization in immunosuppressed organ transplant recipients (and patients) with recurrent skin warts and non-melanoma skin cancer. In this regard, it must also be noted that patients with severe morbidities paid a relevant toll during the COVID-19 pandemic, and this is specifically true for patients with solid organ transplantations and/or immunosuppressive therapies [17]. These patients require frequent screening examinations, including dermatology visits, to reduce the NMSC burden. Therefore, any retrospective analysis of treatment interventions in this field should be analyzed with consideration of local COVID-19 caseloads and regulatory interventions [18].

## 2. Methods

Study design and population: This study is an analysis of a registry of patients treated with the HPV vaccine Gardasil-9^®^ for the secondary prophylaxis of recurrent keratinotic skin lesions at the University Hospital Inselspital Bern in the Department of Nephrology and Dermatology. Patients under immunosuppressive treatment for transplantation or autoimmune disease and patients with cancer and chemotherapy treatment histories were screened. All patients had received three doses of Garasil-9 during or after immunosuppressive or modulatory treatment. Exclusion criteria were as follows: minors (<18 years), incomplete vaccination record, and loss of follow-up after vaccination. Overall, 38 patients were identified; no screened candidates were excluded. Patients were regularly followed and treated for skin lesions based on clinical indications by the treating physician. Visits between 1 year before the first vaccination and 1 year after the last vaccination were analyzed. Patients’ characteristics were collected from electronic health records and laboratory analyses extracted via the Insel Data Science Center (IDSC) and summarized in Table 1. The study protocol was approved by the local ethical committee (ID 2017-01267).

Endpoints and co-variates: The primary endpoint of the study was the difference in the number of visits with major dermatological interventions in the pre-vaccination period (1 year before the first vaccination) compared to the post-vaccination period (1 month after the third vaccination until the end of the study) using patient-id as a grouping variable. Major dermatological interventions were defined as lesion biopsies, punch biopsies, curettages, and excisions. Patients may have experienced multiple interventions at one visit; here, a major dermatological intervention was defined if at least one of the above-defined procedures was performed. Secondary endpoints were differences in visit numbers with only minor interventions (topical treatment and cryotherapies) and overall dermatological visits in the pre- vs. post-vaccination period. As an additional endpoint, we dichotomized patients into responders and non-responders. Patients who had a reduced number of visits with major interventions in the post-vaccination period compared with the pre-vaccination period were defined as responders.

Statistical analysis: Results were reported as the number of participants (percentage) for categorical data and the median (interquartile range) for continuous data. Results were expressed as multivariable-adjusted mean ± SD for categorical values and as adjusted regression coefficients for continuous variables. A two-tailed *p* < 0.05 was considered statistically significant. The primary endpoint was analyzed using a zero-inflated Poisson regression. For the secondary endpoint, a Cox proportional hazard model (PWP gap time model) was employed, using patient_id as a cluster variable and event incidence as a strata variable. Briefly, each patient’s follow-up was segmented at intervals between dermatological visits, and segments were assigned either pre- or post-vaccination status. At each dermatological visit, a dichotomous event was defined: either 0 for none or minor dermatological interventions or 1 for major interventions. Segments between the first vaccination and the last vaccination + 1 month were dropped from the analysis. With the last follow-up within 13 months after the last vaccination, patients were right-censored. All participants were at risk for at least one stratum pre- and one stratum post-vaccination [19]. The PWP model was analyzed in three ways: crude model: time-to-event in dependence on major dermatological intervention (0 or 1) and vaccination status (pre- vs. post-vaccination) as the sole independent predictor. Intermediate model: crude model plus patient age, gender, type of immunosuppression, and type of skin tumor as additional independent variables. Full model: intermediate model plus national-wide COVID-19 prevalence as an additional independent parameter. COVID-19 prevalence accounts for reduced activity in non-vital medical care during the COVID pandemic [17,18]. Overall, a hazard ratio with 95% confidence intervals was calculated for vaccination status as an independent variable for the three different models. In a logistic regression model (GLM), we analyzed the impact of high pre-vaccination interventional burden (top quartile of patients in respect of annual pre-vaccination major interventions), patient age (per year), number of immunosuppressive drugs (per 1), and treatment indication (TPL vs. non-TPL) as independent factors on dichotomous vaccination outcome (responders). Statistical analyses were performed using R (version 4.0.3), tidyverse packages for data analysis, visualization, and survival, and pscl for modeling.

## 3. Results

38 patients who received three doses of the nonavalent HPV vaccine Gardasil-9^®^ between 5 December 2018 and 1 January 2022 due to NMSC and/or recurrent skin warts were identified and included in the study. NMSC included all cSCCs and Cis (intraepidermal carcinoma in situ; this also refers to actinic keratoses and Bowen’s disease). 76% of patients were male; the mean age was 62 years (IQR: 50, 73). 25 (66%) were treated for SOT, 4 (11%) for other cancer (these were: tubal carcinoma, acute myeloid leukemia, diffuse large B-cell lymphoma, polycythemia vera), 2 (5%) for autoimmunity (these were: Crohn’s disease, common variable immunodeficiency (CVID)), and the remainder 7 (18%) for recalcitrant warts. 8 (21%) patients were without immunosuppression, 7 (18%) under monotherapy, 10 (26%) under dual therapy, and 13 (34%) under triple therapy. Among all patients, 16 (42%) received a CNI, 18 (47%) an antimetabolite (Azathioprine or Mycophenolate), 6 (16%) an mTor inhibitor, 17 (45%) PDN, and 9 (24%) other IS (Supplementary Table S1).

Before the study began, 21 (55%) patients were diagnosed with NMSC (15 cSCC, 6 CIS), and 17 (45%) had warts without NMSC. 16 (42%) patients had solitary lesions, and 22 (58%) patients had multiple lesions at sun-exposed localizations (acral, facial). Patient characteristics are summarized in Table 1. All patients received 3 Gardasil-9^®^ vaccinations according to a 3-dose schedule with a median interval of 1.8 months (IQR 1.7–2.1) between the first and second and 6.0 months (IQR 5.6–6.1) between the first and last months. 

Overall, 1040 visits with 915 interventions (422 minors and 493 majors) in 100.0 patient years were analyzed. 318 visits and 285 interventions (102 minor, 183 major) between the first vaccination and one month after the last vaccination were excluded from the analysis. Major interventions included biopsies (n = 69), curettages (n = 538) and excisions (n = 79), minor interventions like cryotherapies (n = 578) and topical treatments (n = 882) (Table 2, Supplementary Table S2). 171 visits were without interventions. 425 visits in 37 patient years were analyzed before vaccination and 297 visits in 38 patient years after vaccination (Table 2).

**Figure 1:**
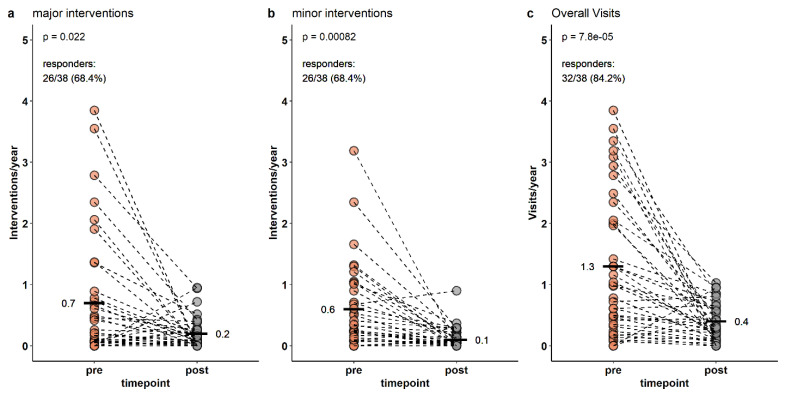
Interventions during study period. Pre: pre-vaccination period from 1 year before first vaccine to first vaccination, post: post-vaccination period from last vaccination + 1 months until end of study. Intervention/year: Number of interventions in the respective period divided by the time of observation. Responders: Patients with a decline of annual intervenions per year.

Visits with major dermatological interventions declined after vaccination from a mean of 0.7/year [IQR: 0.0–0.8] to 0.2/year [IQR: 0.0–0.2] (*p* < 0.05) and 68.4% (26/38) of patients experienced a decline in major interventional events. Additionally, visits with minor interventions declined from a mean of 0.6 [IQR: 0.1–0.9] to 0.1/year [IQR: 0.0–0.1] (*p* < 0.01) with a positive response in similar 68.4% of patients (26/38). Similarly, overall visits declined from 1.3 to 0.4/year (*p* < 0.01), while visits without any interventions remained stable (0.1/year to 0.1/year, *p* = n.s.), see Figure 1.

To further these investigations, we performed a time-to-event analysis for repeated major dermatological events for each patient. Interestingly, hazard radio for major intervention was 0.27 (CI 0.14–0.51, *p* < 0.001) between pre- and post-Gardasil-9^®^. Gardasil-9^®^ intervention remained effective in reducing major dermatological interventions after correction for relevant co-factors (intermediate model, HR 0.2, CI 0.11–0.37, *p* < 0.001) and even after correction for national COVID-case load (full model, HR 0.17, CI 0.09–0.33, *p* < 0.001). A relevant proportion of patients received their vaccination schedule during or in the early phase of the COVID-19 pandemic (21.1% completed their vaccination before, 78.9% after the start of the COVID shutdown in Switzerland on 16 March 2020; Table 3).

Dermatological tumor occurrence is dependent on baseline parameters, including patient age, history of dermatological interventions, treatment regimen, and treatment indication. To study this, we performed logistic regression analysis in a GLM (generalized linear model) with vaccination response (decline of annual visits with major interventions, 26/38 patients) as dependent and previous interventional burden, age at vaccination, number of immunosuppressive drugs, and treatment indication (TPL vs. non-TPL) as independent factors. Indeed, TPL patients (OR 7.1 [CI: 0.83–76.8], *p* = 0.086) and heavy pre-vaccination treatment burden (OR 3.68 [CI: 1.06–17.7], *p* = 0.062) were associated with an increased odds ratio for treatment response. (Table 4).

## 4. Discussion

Cutaneous squamous cell carcinoma and its precursors are the most frequent carcinomas in solid organ transplantation and chronic immunosuppression and correlate with cutaneous human papillomavirus infections, namely the beta-HPV types [1,2,3,4].

The licensed vaccines for HPV immunization are directed against alpha types on mucosa, whereas beta HPV types are mainly found on keratinized skin [3,4]. To date, there is no good evidence on the possible immunizing effect of these vaccinations against antigens from beta-HPVs on keratinized skin. An effective immunological target of HPVs is the viral capsid envelope, consisting of the minor capsid proteins L1 and L2. Therefore, the currently approved vaccines consist of L1 virus-like particles (VLPs) from certain alpha-HPVs. These vaccines also appear to have cross-reactivity with other alpha-HPV subtypes, but this is highly variable and often low-titer [10]. In addition, cross-reactivity has also been measured in an RCT with induced antibody titers of cutaneous beta-HPV types [20]. Although this seroconversion of antibodies against cutaneous HPV was rather low in titer and the variability large in the affected individuals, there is anecdotic evidence that clinical regression of cSCC and its precursors can occur after administration of licensed alpha-HPV vaccination [12,13,14].

We here report a reduction in dermatology interventional treatment burden in IS patients after administration of an alpha-HPV vaccination, namely the Gardasil-9^®^ vaccination. Indeed, median surgical treatment frequency declined by 71% from 0.7/year to 0.2/year within the first 12 months after vaccination. The effect was observed universally throughout various patient groups, involving transplant recipients, older patients, and patients with recurrent diseases, but was most experienced in TPL recipients and patients with a previous high tumor burden.

Notably, the recent COVID pandemic and the shutdown of general medical services had no impact on interventions during the observation period. Patients with a high risk of de novo or recurrent skin tumor burden were not restricted by medical limitations.

However, this study has some limitations. First, the retrospective study design, the lack of comparisons with non-vaccinated patients, and the rather low number of patients do not allow us to exclude all co-founders that may influence the study results (patient no-shows and drop-outs). Furthermore, no detailed analysis of HPV infection (type), no histopathological data, and no data on vaccine-specific HPV antibody measurements before and after vaccination are available.

The question remains whether a possible seroconversion with mostly low titers against non-genital skin HPV types really has clinical relevance. A possible explanation could be a mixed infection of different HPV types. Thus, sometimes genital, mostly high-risk alpha-HPVs are found in cSCC besides beta-HPVs [21,22,23]. Thus, antibodies generated in L1 vaccines could explain some immunity in skin cancer development in selective cases with high mucosal activity [10]. However, large-scale prospective RCTs for definitive confirmation are lacking.

To our knowledge, this is among the largest cohort of dermatology patients at risk for recurrent keratotic skin lesions investigating the effect of Gardasil-9^®^ vaccination in respect of major surgical intervention. The study population comprised a typical cohort of IS patients in a tertiary dermatology clinic and a repeated and well-documented treatment log for vaccination and dermatology interventions.

Furthermore, attempts are being made to develop vaccinations against beta-HPV infections. To date, no anti-beta HPV vaccine is on the market. The large heterogeneity of HPV types in cutaneous keratotic lesions makes the development of efficient anti-beta HPV vaccines difficult [10]. Furthermore, the direct effect of beta-HPV infection on cutaneous carcinogenesis remains obscure. The development of skin cancer has long been considered a co-carcinogenic effect of beta-HPV with UV light [9]. UV irradiation of HPV-infected keratinocytes increases the promoter activity of papillomaviruses, and HPV oncoproteins may inhibit the repair of UV-dependent DNA damage in keratinocytes [9,24]. In addition, immunosuppressive drugs such as calcineurin inhibitors (e.g., ciclosporin A and tacrolimus) have an inhibitory effect on tumor suppressor genes such as p53, which additionally promote the development of skin carcinogenesis, and can therefore also have an inhibitory effect on DNA repair mechanisms in the case of UV damage and HPV infection [9,25]. Another explanation could be the loss of specific T-cell immunity against the papillomavirus. It has been shown in animal models in rats that suppression of a CD8+T cell-mediated immune response to beta-HPV infection promotes the development of cSCCs [10,26]. However, these findings do not explain the increased risk of cSCC development in immunocompetent patients with beta-HPV infection. A potential explanation could be that in certain immunocompetent patients, there is a subclinical dysfunction of specific CD8+ T-cells or a local suppression of cutaneous T-cells by UV light on sun-exposed sides that favors cSCC development [9,10,26].

In summary, the association between beta-HPV and cSCC development and the increasing burden of cSCC development, especially in immunocompromised patients, highlights the need for efficient immunization against HPV-related skin cancer. Although the direct effect of alpha-HPV vaccination on cutaneous HPV infection has not been proven, our study demonstrated a significant decrease in dermatologic procedures in immunosuppressed patients after administration of the Gardasil-9^®^ vaccination. Our data show that with the administration of such a vaccination, the skin tumor burden in these patients can be significantly decreased. This could reduce invasive procedures, overall mortality, and healthcare costs. Our data may pave the way for further prospective and randomized controlled trials, which are needed to prove the effect of alpha-HPV in cutaneous HPV infection.

## Figures and Tables

**Table 1:** Baseline characteristics for patients.

	Patient Characteristics
	N = 38
Age (years)	62 (50, 73)
Sex (male)	28 (76%)
Previous NMSC	21 (55%)
previous cSCC	15 (39%)
Only Wart lesions	17 (45%)
Underlying disease	
TPL	25 (66%)
other cancer	4 (11%)
autoimmune	2 (5%)
recalcitrant warts	7 (18%)
number of IS	
0	8 (21%)
1	7 (18%)
2	10 (26%)
3	13 (34%)
localisation	
solitary	16 (42%)
multiple	22 (58%)

TPL: Transplantation; NMSC: Non-melanoma skin cancer; SCC: Squamous-cell carcinoma; No of IS: number of immunosuppressive drugs.

**Table 2:** Overview of all patient visits and interventions before, during and after Gardasil-9^®^ vaccinations. Before covers a time frame between first visit maximal 1 year before until first vaccination. During covers the time period between first and third vaccination plus 1 month. After covers the time between third vaccination plus 1 month and last visit maximal 1 year after last vaccination.

Variable	Overall	Before	During	After
Visits	1040 [25.5 (IQR: 14–38)]	425 [10 (IQR: 4–16)]	318 [6 (IQR: 3–12)]	297 [5 (IQR: 2.25–12.75)]
Overall interventions	915 [21 (IQR: 10–35)]	394 [8 (IQR: 3–15.75)]	285 [4.5 (IQR: 2.25–11)]	236 [4.5 (IQR: 1–10.75)]
Minor interventions	422 [8.5 (IQR: 2.25–15)]	203 [3.5 (IQR: 1–7)]	102 [1.5 (IQR: 0–3.75)]	117 [1 (IQR: 0–3.75)]
Major interventions	493 [9.5 (IQR: 3.25–19.25)]	191 [2 (IQR: 0.25–6.75)]	183 [1.5 (IQR: 0–6)]	119 [2 (IQR: 0–5.75)]
follow up time	100 [2.7 (IQR: 1.868–3.235)]	37 [0.96 (IQR: 0.712–1.165)]	25 [0.585 (IQR: 0.55–0.608)]	38 [0.765 (IQR: 0.35–1.71)]

**Table 3:** Repeated time to event-analysis for Gardasil-9^®^ Vaccination. ^1^ HR: hazard ratio, ^2^ CI: confidence interval.

	Crude Model			Intermediate Model			Full Model		
	HR ^1^	95% CI ^2^	*p*-Value	HR ^1^	95% CI ^2^	*p*-Value	HR ^1^	95% CI ^2^	*p*-Value
Gardasil	0.27	0.14, 0.51	<0.001	0.21	0.11, 0.41	<0.001	0.2	0.10, 0.41	<0.001
Gender [male]				0.5	0.28, 0.88	0.017	0.5	0.28, 0.88	0.017
Age at vaccionation (per year)				0.99	0.98, 1.00	0.13	0.99	0.98, 1.00	0.13
Indication [TPL]				0.6	0.31, 1.19	0.15	0.6	0.31, 1.16	0.13
Skin lesion [SCC Tumor]				3.69	2.31, 5.89	<0.001	3.68	2.31, 5.86	<0.001
national COVID Cases [per 1000]							1.01	0.99, 1.03	0.5

**Table 4:** Logistic regression analysis for vaccination vaccination response: high intervention burden (top quartile of patients in respect of pre-vaccination major interventions/year). Age at vacc: Patient age at vacination. No of IS: number of immunosuppressive drugs. TPL: Transplantation. ^1^ OR = Odds ratio; ^2^ CI = Confidence Interval.

	Responder Major Interventions			Responder Minor Interventions			Responder Overall Interventions		
	OR ^1^	95% CI ^2^	*p*-Value	OR ^1^	95% CI ^2^	*p*-Value	OR ^1^	95% CI ^2^	*p*-Value
high intervention burden	1.53	1.08, 2.18	0.024	0.76	0.54, 1.08	0.14	1.19	0.94, 1.53	0.2
age at vacc (years)	1	1.0, 1.01	0.4	1	0.99, 1.01	>0.9	1	0.99, 1.00	0.7
no of IS (per 1)	0.95	0.75, 1.22	0.7	1.17	0.93, 1.48	0.2	1	0.84, 1.19	>0.9
indication (TPL)	1.37	0.76, 2.47	0.3	0.7	0.40, 1.23	0.2	1.23	0.81, 1.86	0.3

## Data Availability

Data will be made available on request.

## Supplementary Materials

The following supporting information can be downloaded at: https://www.mdpi.com/article/10.3390/vaccines11091490/s1, Table S1: Immunosuppressive treatments in patients; Table S2: Number of overall interventions in the study population. Patients may have exerienced multiple interventions at one study.

## References

[1] Madeleine M.M., Patel N.S., Plasmeijer E.I., Engels E., Bavinck J.B., Toland A., Green A. (2017). Epidemiology of keratinocyte carcinomas after organ transplantation. Br. J. Dermatol..

[2] Euvrard S., Kanitakis J., Claudy A. (2003). Skin cancers after organ transplantation. N. Engl. J. Med..

[3] Euvrard S., Chardonnet Y., Pouteil-Noble C., Kanitakis J., Chignol M.C., Thivolet J., Touraine J.L. (1993). Association of skin malignancies with various and multiple carcinogenic and noncarcinogenic human papillomaviruses in renal transplant recipients. Cancer.

[4] Nindl I., Rösl F. (2008). Molecular concepts of virus infections causing skin cancer in organ transplant recipients. Am. J. Transplant..

[5] de Jong-Tieben L.M., Berkhout R.J., ter Schegget J., Vermeer B.J., de Fijter J.W., Bruijn J.A., Westendorp R.G.J., Bavinck J.N.B. (2000). The prevalence of human papillomavirus DNA in benign keratotic skin lesions of renal transplant recipients with and without a history of skin cancer is equally high: A clinical study to assess risk factors for keratotic skin lesions and skin cancer. Transplantation.

[6] Harwood C.A., Surentheran T., McGregor J.M., Spink P.J., Leigh I.M., Breuer J., Proby C.M. (2000). Human papillomavirus infection and non-melanoma skin cancer in immunosuppressed and immunocompetent individuals. J. Med. Virol..

[7] Wang J., Aldabagh B., Yu J., Arron S.T. (2014). Role of human papillomavirus in cutaneous squamous cell carcinoma: A meta-analysis. J. Am. Acad. Dermatol..

[8] de Villiers E.M., Fauquet C., Broker T.R., Bernard H.-U., zur Hausen H. (2004). Classification of papillomaviruses. Virology.

[9] Reusser N.M., Downing C., Guidry J., Tyring S.K. (2015). HPV Carcinomas in Immunocompromised Patients. J. Clin. Med..

[10] Gupta R., Rady P.L., Doan H.Q., Tyring S.K. (2020). Development of a β-HPV vaccine: Updates on an emerging frontier of skin cancer prevention. J. Clin. Virol..

[11] CDC (2019). Human Papillomavirus. Epidemiology and Prevention of Vaccine-Preventable Diseases Pink Book.

[12] Nichols A., Nahm W., Rabinovitz H., Ioannides T. (2022). Keratinocyte Carcinomas in Immunocompromised Patients Are Reduced After Administration of the Nonavalent Human Papillomavirus Vaccine. J. Drugs Dermatol..

[13] Nichols A.J., Gonzalez A., Clark E.S., Khan W.N., Rosen A.C., Guzman W., Rabinovitz H., Badiavas E.V., Kirsner R.S., Ioannides T. (2018). Combined Systemic and Intratumoral Administration of Human Papillomavirus Vaccine to Treat Multiple Cutaneous Basaloid Squamous Cell Carcinomas. JAMA Dermatol..

[14] Bossart S., Imstepf V., Hunger R.E., Seyed Jafari S.M. (2020). Nonavalent Human Papillomavirus Vaccination as a Treatment for Skin Warts in Immunosuppressed Adults: A Case Series. Acta Derm. Venereol..

[15] Garbuglia A.R., Lapa D., Sias C., Khan W.N., Rosen A.C., Guzman W., Rabinovitz H., Badiavas E.V., Kirsner R.S., Ioannides T. (2020). The Use of Both Therapeutic and Prophylactic Vaccines in the Therapy of Papillomavirus Disease. Front. Immunol..

[16] Wenande E., Bech-Thomsen N., Togsverd-Bo K., Haedersdal M. (2021). Off-Label 9-Valent Human Papillomavirus Vaccination for Actinic Keratosis: A Case Series. Case Rep. Dermatol..

[17] Bhargava S., Negbenebor N., Sadoughifar R., Ahmad S., Kroumpouzos G. (2021). Global impact on dermatology practice due to the COVID-19 pandemic. Clin. Dermatol..

[18] Elek P., Fadgyas-Freyler P., Váradi B., Mayer B., Zemplényi A., Csanádi M. (2022). Effects of lower screening activity during the COVID-19 pandemic on breast cancer patient pathways: Evidence from the age cut-off of organized screening. Health Policy.

[19] Yang W., Jepson C., Xie D., Roy J.A., Shou H., Hsu J.Y., Anderson A.H., Landis J.R., He J., Feldman H.I. (2017). Statistical methods for recurrent event analysis in cohort studies of CKD. Clin. J. Am. Soc. Nephrol..

[20] Faust H., Toft L., Sehr P., Müller M., Bonde J., Forslund O., Østergaard L., Tolstrup M., Dillner J. (2016). Human Papillomavirus neutralizing and cross-reactive antibodies induced in HIV-positive subjects after vaccination with quadrivalent and bivalent HPV vaccines. Vaccine.

[21] Hama N., Ohtsuka T., Yamazaki S. (2006). Detection of mucosal human papilloma virus DNA in bowenoid papulosis, Bowen’s disease and squamous cell carcinoma of the skin. J. Dermatol..

[22] Iftner A., Klug S.J., Garbe C., Blum A., Stancu A., Wilczynski S.P., Iftner T. (2003). The prevalence of human papillomavirus genotypes in nonmelanoma skin cancers of nonimmunosuppressed individuals identifies high-risk genital types as possible risk factors. Cancer Res..

[23] Ben Ayed I., Tounsi H., Jaballah A., Ardhaoui M., Maaloul A., Lassili T., Mezghani N., Abdelhak S., Boubaker S. (2019). Mucosal human papillomavirus detection and TP53 immunohistochemical expression in non-melanoma skin cancer in Tunisian patients. J. Cutan. Pathol..

[24] Hufbauer M., Lazić D., Akgül B., Brandsma J., Pfister H., Weissenborn S. (2010). Enhanced human papillomavirus type 8 oncogene expression levels are crucial for skin tumorigenesis in transgenic mice. Virology.

[25] Xu J., Walsh S.B., Verney Z.M., Kopelovich L., Elmets C.A., Athar M. (2011). Procarcinogenic effects of cyclosporine A are mediated through the activation of TAK1/TAB1 signaling pathway. Biochem. Biophys. Res. Commun..

[26] Strickley J.D., Messerschmidt J.L., Awad M.E., Li T., Hasegawa T., Ha D.T., Nabeta H.W., Bevins P.A., Ngo K.H., Asgari M.M. (2019). Immunity to commensal papillomaviruses protects against skin cancer. Nature.

