# Supplementary material for: HPV Vaccination in Immunosuppressed Patients with Established Skin Warts and Non-Melanoma Skin Cancer: A Single-Institutional Cohort Study
Source: Vaccines (Basel). 2023 Sep 15;11(9):1490. doi: 10.3390/vaccines11091490 (PMC10535650; doi:10.3390/vaccines11091490)
Supplement: Supplementary file 1 [file vaccines-11-01490-s001.zip › vaccines-2515399-supplementary.pdf]

Supplementary Table S1. Immunosuppressive treatments in patients.

Immunosuppression	n	Frequency
CNI	16	42%
Antimetabolite	18	47%
mTor Inh.	6	16%
PDN	17	45%
Other	9	24%

CNI: Calcineurin Inhibitor. mTor Inh.: mTor Inhibitor. PDN: Prednisolon.

Supplementary Table S2. Number of overall interventions in the study population. Patients may have exerienced multiple interventions at one study visit.

Type	N	Overall	Frq	Intervention
Biopsie	69	2317	3%	major
Curettage	538	2317	23.2%	major
Excision	79	2317	3.4%	major
Kryo	578	2317	24.9%	minor
Topic	882	2317	38.1%	minor
**None**	171	2317	7.4%	none
